# Innate immune responses to duck Tembusu virus infection

**DOI:** 10.1186/s13567-020-00814-9

**Published:** 2020-07-13

**Authors:** Ning Li, Jun Zhao, Yudong Yang, Yongqing Zeng, Sidang Liu

**Affiliations:** College of Veterinary Medicine, Shandong Provincial Key Laboratory of Animal Biotechnology and Disease Control and Prevention, Shandong Provincial Engineering Technology Research Center of Animal Disease Control and Prevention, Sino-German Cooperative Research Centre for Zoonosis of Animal Origin Shandong Province, Shandong Agricultural University, Daizong Street 61, Taian, 271018 Shandong China

**Keywords:** Tembusu virus, Duck, Pathogenesis, Innate immunity, Immune evasion

## Abstract

The disease caused by duck Tembusu virus (DTMUV) is characterized by severe egg-drop in laying ducks. Currently, the disease has spread to most duck-raising areas in China, leading to great economic losses in the duck industry. In the recent years, DTMUV has raised some concerns, because of its expanding host range and increasing pathogenicity, as well as the potential threat to public health. Innate immunity is crucial for defending against invading pathogens in the early stages of infection. Recently, studies on the interaction between DTMUV and host innate immune response have made great progress. In the review, we provide an overview of DTMUV and summarize current advances in our understanding of the interaction between DTMUV and innate immunity, including the host innate immune responses to DTMUV infection through pattern recognition receptors (PRRs), signaling transducer molecules, interferon-stimulated genes (ISGs), and the immune evasion strategies employed by DTMUV. The aim of the review is to gain an in-depth understanding of DTMUV pathogenesis to facilitate future studies.

## Introduction

In April 2010, an outbreak of an unknown duck egg-drop disease occurred in the coastal provinces of southeast China, characterized by a substantial decrease in egg laying and neurological symptoms in infected egg-laying and breeder ducks [1]. At necropsy, hyperemia and hemorrhage of the ovary, follicle atresia and rupture were observed in the diseased ducks. The morbidity rate of this illness was up to 90%, and the mortality varied from 5 to 30% depending on the management conditions. Finally, the duck Tembusu virus (DTMUV) was identified as the causative agent [1]. Tembusu virus was first isolated from Culex mosquitoes as early in 1955 in Malaysia (prototypical strain MM1775) [2], there are few reports associated about this virus until an infectious disease was identified caused by it in a broiler farm in Malaysia in 2000 (named the Sitiawan virus), characterized by encephalitis and retarded growth of chicks [3]. After 10 years with no appearance of the virus, there were large scale outbreaks of Tembusu virus in ducks in the major duck-raising areas of China in 2010, with a rapid spread and huge economic losses [1]. Subsequently, some outbreaks caused by DTMUV in Southeast Asia were reported [4, 5]. Indeed, the increase of emerging and re-emerging infectious diseases are seriously threatening the development of the duck industry [6–14].

Host innate immunity serves as the first line of defense against invading pathogens, and pattern recognition receptors (PRRs), the crucial components of the innate immunity system, can recognize the pathogens, activate the specific signaling cascades, and induce the production of type I interferons (IFN-I) and proinflammatory cytokines, ultimately leading to the establishment of innate immunity and the development of adaptive immunity [15].

Since the outbreak of DTMUV in 2010, comprehensive studies have been performed on the etiology, epidemiology, clinical symptoms, and pathology of the virus. However, the research on the molecular pathogenesis of DTMUV is still at the developmental stage. The complex interactions between the virus and host immune response are critical for understanding the virus pathogenesis. In recent years, much work has been done in this area, especially research on host innate immune responses induced by DTMUV infection. In this review, the advances in the study of DTMUV and its interaction with host innate immunity are summarised, which can facilitate future studies on DTMUV pathogenesis and provide new insights into the prevention and treatment of the disease.

## Overview of DTMUV

DTMUV is an enveloped, positive sense, single-stranded RNA virus belonging to the genus Flavivirus in the family Flaviviridae, which also includes West Nile virus (WNV), dengue virus (DENV), Japanese encephalitis virus (JEV), and Zika virus (ZIKV) [1]. The DTMUV genome is approximately 11 kb, encoding three structural proteins (capsid, C; pre-membrane, PrM; and envelope, E) and seven non-structural proteins (NS1, NS2A, NS2B, NS3, NS4A, NS4B, and NS5) [16]. The DTMUV strains are virulent and have strong pathogenicity in ducks, causing systemic infection. The infected ducklings display retarded growth and neurological symptoms, while laying ducks display a severe drop in egg production [1]. Various breeds of ducks can be infected with DTMUV, and age-related differences in susceptibility to the virus were significant. Several studies showed that the susceptibility of ducklings and goslings to DTMUV gradually decreased with increasing age within 7-week-old ducks [17–19], but in different week-old breeding ducks, 14- to 16-week-old reserve breeding ducks were more resistant to DTMUV than 55-week-old egg-laying ducks [20, 21]. The increasing pathogenicity and expanding host range of emerging DTMUV strains are observed: the virus can infect not only ducks, but also chickens and geese [22]. Moreover, DTMUV replicates well in several mammalian cell lines, such as BHK-21, Vero, and 293T cells, and it can even infect mice under artificial conditions [23, 24].

DTMUV has strong transmission ability, which can spread among ducks through contact and vertical transmission [25]. Notably, Li et al. reported that DTMUV can also be transmitted by aerosol [26], similar to H9N2 avian influenza virus (AIV) [27, 28], Newcastle disease virus (NDV) [29, 30], and Marek’s disease virus [31]. This transmission mode partly explains why DTMUV was capable of spreading to majority of duck-raising areas in China within a short time after the outbreak. Many members of the genus Flavivirus are arboviruses. Tang et al. isolated a mosquito-origin Tembusu strain from duck farms with known DTMUV outbreaks [32], and a Tembusu strain was also isolated from house sparrows living around the poultry farms, suggesting that DTMUV can be spread not only by mosquitoes, but also by sparrows [33]. A large number of serological and etiological detection methods has been established since the DTMUV outbreak, including ELISA [34], neutralizing antibody detection [35], quantitative real-time PCR (qPCR), and reverse-transcription loop-mediated isothermal amplification (RT-LAMP) [36, 37]. The common molecular diagnostic methods for DTMUV were compared by previous studies, in which the RT-LAMP and qPCR usefulness for rapid diagnosis was demonstrated, especially the former was more useful in DTMUV field detection [38]. Based on the various detection methods, a large amount of epidemiological information on DTMUV was obtained, and the results showed that co-infection with DTMUV, H9N2 AIV, and NDV was common [39], and DTMUV infection may escalate the avian pathogenic *Escherichia coli* incidence in ducks [40]. The phylogeographical analysis indicated that current DTMUV strains circulating in Asia are genetically classified into 3 clusters, including cluster 1, cluster 2 (2.1 and 2.2) and cluster 3 [41].

In animal experiments, qPCR demonstrated that the load of DTMUV in the spleen was higher than in other organs in early infection [17, 42]. The virus could last from 2 hours post infection (hpi) to 18 days post infection (dpi) in the spleens of egg-laying shelducks. Furthermore, DTMUV particles were observed mostly in lymphocytes and macrophages by transmission electron microscope analysis [43]. Recently, Ma et al. verified that monocytes/macrophages were the key targets of DTMUV infection [44]. Therefore, the viral load in the spleen first rapidly increases after TMUV infection, which provides a good cell model for in-depth study of viral pathogenesis.

It has been reported that endocytosis through endosomes is an efficient mechanism used by many viruses to break through the physical barrier of the cellular plasma membrane to enter the cell and initiate productive infection. Normally, flavivirus entry occurs by receptor-mediated endocytosis [45]. Heat shock protein A9 and glycoregulatory protein 78 have been identified as binding receptors for DTMUV in DF-1 cells [46, 47], and clathrin-mediated endocytosis was also necessary for DTMUV entry into BHK-21 cells. The acidic pH in the endosome induced structural alterations in the viral E protein, leading to membrane fusion and uncoating [48]. Therefore, the viral RNA genome was translated to initiate virus replication, at the same time the ubiquitin-proteasome system also played an important role in DTMUV replication [49]. In addition to mediating virus entry, E protein is essential for DTMUV pathogenesis [50]; especially, mutations in several important amino acid sites, which can significantly affect viral pathogenicity. Yan et al. reported that a single mutation at amino acid residue 156 (S-P) reduced the ability of viral replication and transmission in ducks, and further analysis confirmed that the potential mechanism was composed by the disruption of N-linked glycosylation at position 154 and changes in the conformation of the “150 loop” of the E protein [51]. Recently, it has been found that the threonine-to-lysine mutation of residue 367 in E protein can attenuate DTMUV [52]. As research continues, the effects of other proteins on viral replication will be discovered.

To date, the categories of DTMUV vaccine are various, including inactivated vaccines [53, 54], attenuated live vaccines [55, 56], and DNA vaccines [57–59]. This disease still occurs in some duck farms due to lack of immunization or immunization failure, although there are several commercial inactivated and attenuated live vaccines in China. Considering that many flaviviruses such as WNV, DENV, and JEV are pathogens of zoonoses, the positive antibodies of DTMUV were detected in duck farm workers [60], DTMUV may be a potential threat to public health. Therefore, more attention should be paid to epidemiological investigation and evolution analysis.

## DTMUV infection triggers host innate immune responses

Innate immune responses are required to protect the host from pathogenic infections in the early stages. PRRs mainly comprise five family members: toll-like receptors (TLR), retinoic acid-inducible gene I (RIG-I)-like receptors (RLR), nucleotide binding oligomerization domain (NOD)-like receptors (NLR), C-type lectin receptors (CLR), and absent in melanoma 2 (AIM2)-like receptors (ALR). The different PRRs in the cell membrane, endosome, and cytoplasm can sense various pathogen-associated molecular patterns (PAMPs) such as the RNA and DNA of viruses, lipopolysaccharide and peptidoglycan of bacteria, etc. Upon activation of PRRs, they will interact with the specific adaptor proteins, resulting in activation of immune signaling pathways and establishment of innate immunity characterized by the induction of the IFN-I, antiviral molecules, and inflammatory cytokines [15, 61]. To date, studies on the interaction between DTMUV and innate immunity have increased.

### TLR-mediated signaling pathway in recognition of DTMUV

TLR, a group of conserved type I transmembrane proteins, is one of the most critical PRRs which can sense the different invading pathogens outside the cell membrane and internally in endosomes and lysosomes. Currently, 10 TLR have been reported in human, and 10 TLR in chicken, while only 5 TLR (TLR 2 [62], TLR3 [63], TLR4 [64], TLR5 [65], and TLR7 [66]) in duck. Furthermore, there are some differences between avian and mammal TLR, including the absence of TLR8 and TLR9 and the presence of TLR1La, TLR1Lb, TLR15, and TLR21 in chickens [67, 68]. TLR7 recognizes single-stranded RNA (ssRNA), Pekin duck TLR7 is most highly expressed in the spleen, bursa of Fabricius, and lung, sharing 85% identity with chicken TLR7 and 66% with human based on amino acid alignment [66]. Muscovy duck TLR3 shares 87.3% amino acid identity with chicken and 62% with human, with a broad expression spectrum [63]. TLR3 detects double-stranded RNA (dsRNA) and elicits responses.

**Figure 1:**
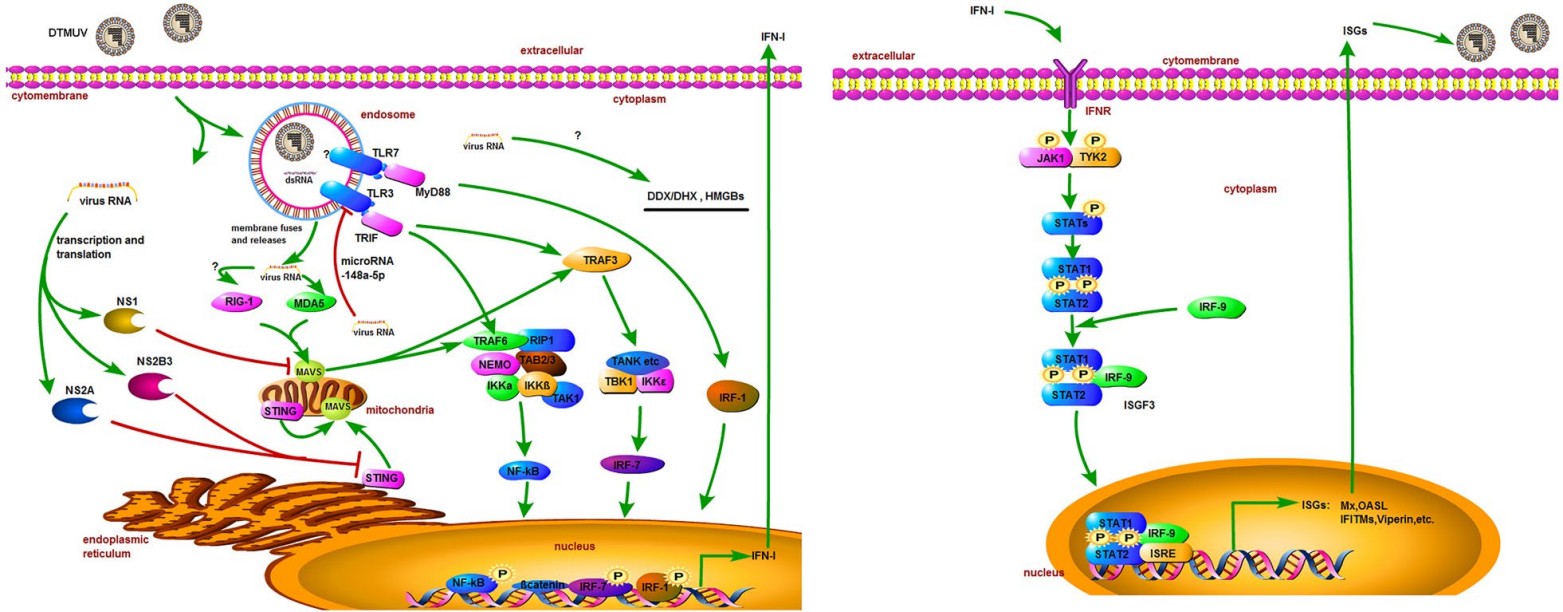
**Duck Tembusu virus (DTMUV) infection triggers the innate immunity signaling pathways in the cell.** Both MDA5 and TLR3 can recognize DTMUV and elicit signaling cascades through adaptor proteins MAVS and TRIF, respectively, leading to the induction of IFN-I production. The JAK-STAT signaling pathways mediated by IFN-I induce the abundant expression of various ISGs, including Mx, OASL, IFITMs, Viperin etc., and they are capable of inhibiting DTMUV replication. However, the specific role of other PRRs such as RIG-I, TLR7, DDX/DHX, and HMGBs is unclear, it needs further study. To date, DTMUV has evolved several strategies to escape from host immune responses. The protein NS1 of DTMUV can bind MAVS to impair the IFN-I expression level. NS2A and NS2B3 target the transcription molecule STING to inhibit the IFN-I, resulting in the immune evasion. Additionally, DTMUV may downregulate the expression of TLR3 by microRNA-148a-5p.

Activated TLR3 and TLR7 recruit their individual adaptor protein, called Toll-interleukin (IL)-1-resistance (TIR) domain-containing adaptor inducing IFN-β (TRIF) and myeloid differential protein-88 (MyD88), respectively [69, 70]. TRIF activation leads to the formation of the complex of tumor necrosis factor (TNF) receptor-associated factor 6 (TRAF6) [71], receptor-interacting protein 1 (RIP1), the TAK1-binding proteins 2 (TAB2) and TAB 3, and the NF-kB essential modifier (NEMO) [72]. Eventually, NF-kB is activated and enters the nucleus after ubiquitination and degradation of IκB. At the same time, TRIF recruits TRAF3, in this period, TRIF interacts directly with TRAF family member-associated NF-κB activator (TANK) and TANK-binding kinase 1 (TBK-1) [73], as well as the kinases TBK-1 and IKKε phosphorylate IFN regulatory factor (IRF) 7. Ultimately, activated NF-κB and IRF7 induce the production of IFN-I and inflammatory cytokines, leading to the establishment of the innate immune response. However, duck TLR7-MyD88 signaling pathway triggers IFN-I production through the downstream molecule IRF-1 (Figure 1).

In our previous study, DTMUV infection can stimulate the expression of a large number of innate immune-related genes in the brain and spleen of Cherry Valley ducks. According to the qPCR analysis, the expression of TLR3 increased 28.54- and 1.57-fold at 2 dpi in brain and spleen, respectively [74]. The TLR3 expression was also measured in the brain, liver and spleen of goslings infected with DTMUV, and it was found that TLR3 upregulated most significantly compared to other PRRs (88.43-fold in brain, 57.79-fold in liver, and 12.58-fold in spleen) [75]. The expression of PRRs in chicken embryo fibroblasts (CEF), 293T cells, and chicks were detected by Chen et al. after DTMUV infection by qPCR at the indicated time. The results showed that DTMUV could significantly upregulate transcript levels of TLR3, but its effect on the expression of TLR1, TLR2, TLR5, TLR7, TLR15, or TLR21 in CEF cells is relatively low. Moreover, in the 293T cells which can stably express shRNA targeting TLR3, the functions of TLR3 in innate immune during DTMUV infection were confirmed, suggesting that DTMUV can efficiently trigger TLR3-dependent signaling pathways [76]. Increasing intracellular signal transducers were identified, numerous studies showed that many proteins could be involved in DTMUV infection. For example, the TRIF in duck embryo fibroblasts (DEF) cells infected with DTMUV was upregulated [77], duck TRAF3 can interact with the upstream molecule TRIF, and leading to the production of IFN-β, overexpression of TRAF3 inhibited the replication of DTMUV [78], while the overexpression of duck TBK1 and IRF7 dramatically reduced the replication of DTMUV in DEF cells [79–81]. The results above clearly demonstrated that a TLR3-mediated signaling pathway was essential for defending against DTMUV infection.

In addition to TLR3, other TLRs were found to be involved in the recognition of DTMUV through transcriptomics and proteomics analysis. Yu et al. analyzed the transcriptomic data of DTMUV-infected DEF cells collected at 12 and 24 hpi, and they found that DTMUV significantly downregulated expression of TLR5, while the expression of TLR7 was upregulated [82]. There is a RNA-sequencing study of goslings infected with DTMUV, in which only TLR7 experienced a significantly upregulated trend, and qPCR verified that the change of TLR7 in the liver (29.15-fold) is much higher than in the spleen (2.85-fold) and brain (3.24-fold). More importantly, the signaling proteins MyD88, TRAF3, and NF-κB were significantly upregulated [75], and DTMUV infection significantly increased the expression of IRF-1, indicating that the virus might induce MyD88 signaling pathway [83]. Differentially expressed proteins in DTMUV-infected BHK-21 cells were quantitatively identified by Sun et al. by iTRAQ, the production of TLR9 increased at 48 hpi [84], which suggested that TLR9 can be potentially implicated in DTMUV infection in mammalian cell lines.

It has been showed in previous studies that the TLR3, TLR5, and TLR7 involved in DTMUV recognition can be changed by flavivirus infection [85–87]. However, whether these PRRs-mediated signaling pathways have positive effect on defending against DTMUV in vivo is uncertain, because the absence of some PRRs is beneficial to host survival. For example, TLR3-deficient mice can survive longer than wild mice following lethal influenza virus infection, because of the reduced pro-inflammatory cytokine responses (called “cytokines storm”) which caused damage and pathology to the host [88, 89]. Strikingly, the robust production of pro-inflammatory cytokines was observed during DTMUV infection in vivo and in vitro [74, 82]. Further study is needed on the effect of pro-inflammatory cytokines such as interleukin (IL) 6 and chemokine 8 during viral infection.

### RLR-mediated signaling pathway in recognition of DTMUV

RLR family is constituted by RIG-I, melanoma differentiation-associated gene 5 (MDA5), and Laboratory of genetics and physiology 2 (LGP2). RIG-I and MDA5 are expressed in the cytoplasm and primarily recognize 5′-triphosphate ssRNA and dsRNA [90, 91]. The protein structures of RIG-I and MDA5 are similar, both of them possess tandem caspase activation and recruitment domain (CARD) at N-terminal, followed by a central DExD/H box RNA helicase domain and a repressor domain at C-terminal [92], but the protein structure of LGP2 is different from RIG-I and MDA5′s, which lacks the CARD and is generally considered to be a negative regulator of IFN production [93]. Upon recognition of ligands, RLR recruits and interacts with the mitochondrial antiviral-signaling protein (MAVS) adaptor protein via CARD, and then the activated MAVS triggers a complex signaling transduction pathway, which is similar to the events described downstream of TRIF in Figure 1. Finally, the activation of NF-κB and IRF7 stimulate the expression of IFN-I [92].

It has been observed that RIG-I and MDA5 were involved in the host innate immune response to DTMUV in ducks. The importance of MDA5 might be more than RIG-I based on the degree of changes, and the roles they played might differ with time and tissues [74, 75]. Comparative transcriptomic analysis showed that MDA5 and RIG-I respectively increased 19.76 fold and 17.52 fold, at 24 hpi in DEF cells infected with DTMUV, and transcription factors NF-κB and IRF-7 also showed upregulation [82]. DTMUV could significantly upregulate the expression of MDA5 in CEF cells, and while in the DTMUV-infected 293T cells lack MDA5, the expression of IFN-β expression decreased. It is also demonstrated that the production of IFN-β in 293T cells reduced significantly since MAVS, IRF3, IRF7, and NF-κB are disrupted, they were all required in DTMUV-induced upregulation of IFN-I [76]. Overexpression of duck MAVS significantly reduced DTMUV replication, its disruption increased virus titer in DEF cells [94], and the knockdown of MAVS impaired the activation of IRF1, NF-κB, and IFN-β induced by DTMUV [95], which suggested that DTMUV can efficiently trigger RLR- and MAVS-dependent signaling pathways. LC–MS/MS was used to analyse quantitative proteomic of ovarian follicles collected from shelducks infected with DTMUV, the KEGG analysis of differential expression proteins showed that RLR signaling pathways were involved in DTMUV infection [96]. Additionally, the RIG-I, MDA5, LGP2, and stimulator of interferon gene (STING) were all significantly increased at 5 dpi in goslings infected with DTMUV, especially LGP2, which changes most based on transcriptome data [75]. Except for this study, there are few studies on the role of LGP2 in DTMUV infection, while the LGP2 genes of duck and goose were successively identified, and it is also demonstrated that the mRNA expression levels were significantly upregulated in the brain, spleen and lung after H5N1 AIV infection [97, 98]. Therefore, the specific role of LGP2 in DTMUV infection may be revealed in future studies.

### Other PRRs relevant to DTMUV infection

In addition to TLR and RLR, other PRRs including DExD/H-box RNA helicase family, NLR, and high-mobility group box protein (HMGB) also play crucial roles in response to microbial infection. DExD/H-box RNA helicase has many members, including RIG-I and MDA5. Furthermore, DDX/DHX proteins with conserved helicase domains but lacking the CARD, belong to this family as well, such as DDX1, DDX3 and DDX5. DDX proteins, which can regulate the replication of many viruses [99, 100]. Recently, several studies demonstrated the expression changes of several DDX proteins after DTMUV infection. Sun et al. reported that DDX3X and DDX5 were significantly decreased in DTMUV-infected BHK-21 cells, and they found that DDX3X overexpression could inhibit DTMUV propagation by modulating the IFN-I via TBK1 protein [84], which was consistent with results showing duck DDX3X affecting DTMUV replication [101]. However, other quantitative proteomic analyses revealed that DDX5 was upregulated (1.84 fold) in DEF cells infected with DTMUV [102]; the result was different from DDX5 detection in mammalian cells, indicating that there might be a difference in the innate immune responses to DTMUV between avians and mammals. Additionally, DDX42, DDX60, DHX15, and mov10 RISC complex RNA helicase were differentially expressed after DTMUV infection [82, 102], suggesting that these helicase proteins should be involved in DTMUV infection, but the specific roles were unclear.

In general, NLR was found to sense bacterial PAMP, but in the goslings infected with DTMUV, NOD1 was upregulated (3.57 fold in the spleen, 6.45 fold in the liver, and 3.11 fold in the brain), and its downstream CASP1, CARD9 and others in NOD-like pathways also showed expression differences [75]. Meanwhile, NLRP3 and NLRC5 were found to be involved in DTMUV infection [82]. HMGB proteins including HMGB1 and HMGB3 have been reported to affect DTMUV replication as well [102, 103]. Although various PRRs might be involved in DTMUV infection, only the expression changes of these PRRs were focused on, and their specific function needs further investigation.

### IFN-stimulated genes (ISGs) defense against DTMUV infection

As is well known, IFN-I plays a pivotal role in innate immune responses to viral infection. The synthesized IFN have been secreted, which can bind to specific receptors on neighboring uninfected cells. This leads to the activation of the receptor associated tyrosine kinases JAK1 and TYK2, and therefore phosphorylates signal transducers and activators of transcription (STAT) 1 and STAT2. The phosphorylated STAT1 and STAT2 proteins combine with IRF9 to form IFN-stimulated gene factor 3 (ISGF3), this complex translocates to the nucleus to promote an abundant expression of IFN-stimulated genes (ISGs), which encode distinct antiviral proteins [104, 105]. There are several studies demonstrating that duck JAK1 and TYK2 kinases exhibited antiviral activity against DTMUV infection, and knockdown of two signal transducers significantly inhibited DTMUV-induced IFN-stimulated response element promoter activation [106, 107]. Meanwhile, DTMUV infection can upregulate the STAT1 transcript level [82], indicating that JAK-STAT signaling pathway can defend against DTMUV by inducing the production of hundreds of ISGs [75]. In this review, the advances in interaction between some ISGs and DTMUV were summarised.

#### Mx

Myxovirus resistant (Mx) protein serves as an antiviral molecule and is produced by various cells such as hepatocytes, endothelial cells, and immune cells [108]. The Mx mRNA level was significantly upregulated in DTMUV-infected DEF cells [82]. Hu et al. reported that Mx protein increased by 24.43 fold in DEF cells at 42 hpi [102], and goose Mx was identified as the key in the inhibition of DTMUV replication by transcriptomic analysis. At the same time, the overexpression and knockdown assay in vitro further confirmed that the Mx played crucial roles in the anti-DTMUV effect of IFN signaling pathways [109]. Further mechanism research of Mx demonstrated that two amino acids at the 125 (Lys) and 145 (Thr) positions in the GTP-binding domain were vital for the antiviral function of Mx against DTMUV [110].

#### OASL

The 2′-5′-oligoadenylate synthase (OAS) family proteins belong to a nucleotidyltransferase superfamily [111]. There are four OAS family members in mammals: OAS1, OAS2, OAS3, and OAS-like (OASL) [112], while in poultry only OASL was identified [113, 114]. All of these four OAS members have an NTase domain at N-terminal and one–three OAS domain, but OASL has two ubiquitin-like (UBL) domains at C-terminal, which is different from others. OAS family proteins demonstrate extensive antiviral ability, which can act against many viruses such as DENV, WNV, and JEV, through RNase L-dependent and RIG-I-dependent signaling pathways [112]. In DTMUV-infected DEF cells, the mRNA and protein levels of OASL were significantly increased [82, 102]. The antiviral activity assays of duck OASL showed that OASL overexpression slightly inhibited DTMUV replication, whereas OASL knockdown increased viral replication in DF-1 cells [114]. A study found that DTMUV infection could significantly upregulate the mRNA expression level of goose OASL in vivo [113], and the further study showed that goose OASL could efficiently inhibit DTMUV replication in vitro, while has been proved to be independent of OAS enzyme activity and the UBL domains [115]. Recently, Rong et al. demonstrated that duck OASL inhibited the replication of a set of RNA viruses, such as influenza virus and NDV, by activating and magnifying the OAS/RNase L pathway in a UBL-dependent manner. Functional analysis indicated that three aspartic acid residues in the N-terminal were very important for the switching of avian and mammalian OASL to activate the OAS/RNase L and OASL/RIG-I pathways [116]. However, whether or not duck OASL can efficiently inhibit DTMUV replication, the underlying signaling pathway and molecular mechanism utilized by duck OASL should be further explored.

#### IFITM

Interferon-inducible transmembrane (IFITM) proteins are a subfamily of large transmembrane proteins consisting of IFITM1, IFITM2, IFITM3, IFITM5, and IFITM10. IFITM1, IFITM2, and IFIMT3 are generally considered to be involved in antiviral immunity [117, 118]. IFITM proteins are known to restrict the replication of a large number of viruses by blocking viral entry, including restriction of virus fusion with the late endosomal or lysosomal and penetration into the cytoplasm [119, 120]. IFITM1 and IFITM3 have been recently identified in goose. Goose IFITM3 was activated in goose peripheral blood mononuclear cells infected with DTMUV or treated with TLR agonists. Both IFITM1 and IFITM3 were upregulated in the sampled tissues after DTMUV infection, especially the cecum and cecal tonsil, where DTMUV was intensively located, indicating that DTMUV is responsible for the high expression levels of goose IFITM1 and IFITM3. Notably, the lowest viral copy numbers were seen in the lung, where a high expression of IFITM3 and IFN-I appeared. These data suggested that goose IFITM1 and IFITM3 might positively facilitate IFN-mediated defenses against DTMUV [121]. Similar conclusions were drawn by the investigation of Chen et al., they revealed that DTMUV infection induced robust expression of IFN-I, IFN-III, and IFITMs in vivo and in vitro. It is also demonstrated that the disruption expression of endogenous IFITM1 or IFITM3 markedly enhanced DTMUV replication in DF-1 cells while IFITM2 not, and the overexpression of chicken or duck IFITM1 and IFITM3 can inhibit the replication of DTMUV in DF-1 cells, which indicated that in IFITM family proteins, IFITM1 and IFTIM3 play crucial roles in anti-DTMUV infection [122].

#### IFIT5

Interferon-induced protein with tetratricopeptide repeats 5 (IFIT5) protein serves as an ISG in host innate immunity, and it is also an important adaptor protein, bridging RIG-I to MAVS to enhance RLR signaling pathways [123, 124] and upregulating NF-κB to promote IFN production [125]. Most mammals have four members: IFIT1, IFIT2, IFIT3, and IFIT5. However, IFIT5 is the single member found in birds [126]. Tissue distribution analysis demonstrated that duck IFIT5 was ubiquitously expressed in tissues of 5-day-old ducklings, with the highest expression in the heart, followed by the thymus, cerebrum, liver, and lung [127]. The recombinant duck IFIT5 inhibited the replication of highly pathogenic H5N1 AIV in DF-1 cells [128]. Several differentially expressed proteins involved in the immune response were observed between DTMUV-infected and control duck ovarian follicles. Of these proteins, the upregulation of IFIT5 and OASL was validated at the mRNA and protein levels [96], suggesting both proteins might be involved in the immune response to DTMUV in duck ovarian follicles.

#### Viperin

Viperin, also known as RSAD2 and cig5, is a highly conserved antiviral protein involved in innate immunity [129]. It has been confirmed that viperin protein has a broad antiviral effect on a wide range of viral pathogens such as human immunodeficiency virus [130], influenza virus [131], and hepatitis C virus (HCV) in vitro and in vivo [132]. Duck viperin can be strongly induced by NDV infection in vitro and in vivo [133], and it can be induced by DTMUV infection in DEF cells [82, 102]. Zhu et al. revealed that the overexpression of duck viperin reduced DTMUV replication by inhibiting viral budding in BHK-21 cells. In their study, the binding proteins complex was determined by mass spectrometry, and six proteins were found, including DDX3X and DDX5, which might be involved in the inhibition of DTMUV, possibly indicative of a viperin anti-DTMUV pathway in ducks [134]. These results reflect the importance of DDX3X and DDX5 during DTMUV infection as well.

In addition to the well-known ISGs described above, there are many other ISGs like double-stranded RNA-dependent protein kinase (PKR) and zinc finger CCCH-type antiviral protein induced expression changes by DTMUV infection investigated by many some transcriptomic and proteomic studies [82, 102], and they all have strong antiviral ability [135, 136]. However, whether these ISGs are capable of impairing DTMUV infection is not clear.

## The strategies of DTMUV escape from innate immunity

The previous literature demonstrated that the DTMUV infection can elicit intense immune responses, but circumventing host antiviral innate immune barriers to establish a successful infection is firstly needed [44]. There are several evasion strategies has been evolved by DTMUV to disrupt innate immunity and facilitate productive infection.

### The effect of DTMUV on PRRs expression

As described above, MDA5- and TLR3-dependent signaling pathways can induce large amounts of IFN-I and ISGs to resist DTMUV infection. Exosomes, which are small membrane vesicles formed by the inward budding of the plasma membrane in endocytosis, play a critical role in innate immunity and intercellular communication [137]. In the study of Guo et al., microRNA (miRNA)-148a-5p in DEF derived exosomes could target TLR3 and downregulate the expression of TLR3 and IFN-β, indicating that this miRNA is a negative regulator of TLR3, and impairs the TLR3-mediating innate immune response. The result suggested that DTMUV might inhibit PRRs by inducing miRNA expression to achieve immune evasion, although the expression of miRNA148a-5p was decreased partially due to the inhibition of highly expressed TLR3 in DEF cells and DEF-derived exosomes infected with DTMUV [138]. As far as we know, this is the only study on DTMUV regulating PRRs expression. However, the V protein of most paramyxoviruses can directly interact with MDA5 and inhibit its activity [139–141]. Whether proteins of DTMUV can directly or indirectly interact with MDA5 or TLR3 remains unclear.

### Inhibition of IFN production by targeting important adaptor molecules

Adaptor proteins are essential for TLR- and RLR-mediated induction of IFN-I. The overexpression of MAVS, TRIF, TRAF3, NF-κB, and IRFs can promote the production of IFN-I to inhibit DTMUV replication. However, DTMUV can inhibit IFN-β induction during early infection by targeting several adaptor proteins. Wang et al. reported that the NS1 protein of DTMUV could target the adaptor protein MAVS, and disrupt the interaction with MAVS and RIG-I/MDA5, leading to inhibition of IFN-I expression [142]. In addition to MAVS, STING is also an important adaptor protein, it activates the IRF-3 and NF-κB to stimulate IFN-I production and is necessary for early efficient induction of IFN-I mediated by RLR [143, 144]. It has been demonstrated in the nowadays studies that both NS2A protein and NS2B3 polyprotein of DTMUV can inhibit IFN-β induction by interacting with the STING protein, but the specific mechanism was different. The NS2A protein and TBK1 competed binding to duck STING, disrupted STING dimer formation and reduced TBK1 phosphorylation, leading to the inhibition of IFN-β production. The amino acid residues at 164, 167, and 361 in STING were important for NS2A binding with STING [145]. However, NS2B3 polyproteins inhibited IFN induction by hydrolyzing duck STING, and further mapping analysis showed the scissile bond located between the R84 and G85 residues of STING [146]. It is suggested by the immune evasion strategies employed by DTMUV via targeting MAVS and STING were critical for host innate immune responses to DTMUV infection.

Similarly, the Zika virus circumvented host innate immunity with NS3 and NS2B3 respectively targeting MAVS and STING [147]. The NS3/4A protein of HCV cleaves the MAVS and TRIF proteins, leading to the blocking of RLR and TLR3 signaling [148, 149]. The flaviviruses have evolved many strategies to escape host immune responses, including delaying PRR detection during the early stages of infection, inhibition of IFN gene transcription, and suppression of IFN signaling. Especially there are several non-structural proteins of flaviviruses can efficiently impair the JAK-STAT signaling pathway by targeting the signal transducers [150]. Therefore, we speculate that DTMUV may be capable of escaping immune responses by other approaches, especially the effect of DTMUV on the JAK-STAT signaling pathway.

## Conclusions and perspectives

Since 2010, the studies of the disease associated with DTMUV have made great progress, especially on etiology, epidemiology, diagnostic methods, and immune prevention and control. Recently, as an increasing number of immune molecules were available in the innate immune system of ducks, there is a deeper understanding on the interaction between DTMUV and duck innate immunity. To summarize, a large number of innate immune-related genes, including PRRs, transcription molecules, IFN-I, and ISGs, are significantly upregulated after DTMUV infection in vivo and in vitro. Meanwhile, there are solid and reliable experimental results showing that MDA5- and TLR3-mediated signaling pathways induced the production of IFN-I to defend against DTMUV infection and various ISGs such as Mx, OASL, IFITM1, and IFITM3 can inhibit the replication of DTMUV. It is notable that DTMUV has developed several strategies during its adaptive evolution to circumvent host innate immunity, which should be our focus. Researches on evasion mechanisms of DTMUV also provide scientific reference for the related study of zoonotic viruses in the flavivirus genus.

However, despite great progress being made, there are still many problems worth further exploration, for example: whether other PRRs can recognize DTMUV through studies on other TLR and NLR and their adaptor molecules in ducks during DTMUV infection, the specific mechanism of the up-regulated ISGs in regulating DTMUV replication, the roles of the neglected downregulated proteins and pro-inflammatory cytokines including TLR5, DDX3X, IL-6, IL-12, IL-28, and IL-29 in defending against DTMUV infection, the interaction between individual protein of DTMUV and innate immunity, and whether there are more immune evasion strategies employed by DTMUV. We believe that with the development of research, these questions will be addressed, and the understanding of DTMUV disease will be deepened. Certainly, the purpose of interaction research is to elucidate the pathogenicity of DTMUV, to explore more immune mechanisms such as antiviral signaling pathways and antiviral proteins, and finally to translate these findings to the clinic to prevent and treat this disease.
